# The Role of CEP55 Expression in Tumor Immune Response and Prognosis of Patients with Non-small Cell lung Cancer

**DOI:** 10.34172/aim.2022.72

**Published:** 2022-07-01

**Authors:** Haiyin Fan, Jin Zhang, Bin Zou, Zhisheng He

**Affiliations:** ^1^Thoracic Department, Jiangxi Cancer Hospital, Nanchang, Jiangxi, China; ^2^Ultrasound Department, Jiangxi Chest Hospital, Nanchang, Jiangxi, China

**Keywords:** CEP55, Microarray, NSCLC, Prognostic Model, Tumor Immune Response

## Abstract

**Background:**

With the continuous advancement of diagnostic methods, more and more early-stage Non-small cell lung cancer (NSCLC) patients are diagnosed. Although many scholars have devoted substantial efforts to investigate the pathogenesis and prognosis of NSCLC, its molecular mechanism is still not well explained.

**Methods:**

We retrieved three gene datasets GSE10072, GSE19188 and GSE40791 from the Gene Expression Omnibus (GEO) database and screened and identified differentially expressed genes (DEGs). Then, we performed KEGG and GO functional enrichment analysis, survival analysis, risk analysis and prognosis analysis on the selected hub genes. We constructed a protein-protein interaction (PPI) network, and used the STRING database and Cytoscape software.

**Results:**

The biological process analysis showed that these genes were mainly enriched in cell division and nuclear division. Survival analysis showed that the genes of CEP55 (centrosomal protein 55), NMU (neuromedin U), CAV1 (Caveolin 1), TBX3 (T-box transcription factor 3), FBLN1 (fibulin 1) and SYNM (synemin) may be involved in the development, invasion or metastasis of NSCLC (*P*<0.05, logFC>1). Prognostic analysis and independent prognostic analysis showed that the expression of these hub gene-related mRNAs was related to the prognostic risk of NSCLC. Risk analysis showed that the selected hub genes were closely related to the overall survival time of patients with NSCLC.

**Conclusion:**

The DEGs and hub genes screened and identified in this study will help us to understand the molecular mechanisms of NSCLC, and CEP55 expression affects the survival and prognosis of patients with NSCLC, and participates in tumor immune response.

## Introduction

 Non-small cell lung cancer (NSCLC) is the most common lung cancer. It has an increasing incidence, and most patients are already at an advanced stage by the time of diagnosis^1^; thus, early diagnosis and treatment of NSCLC are very important. There are many risk factors for NSCLC, among which tobacco and air pollution are the most important. Other factors include occupational hazards, as well as dietary and genetic factors.^2,3^ The latest Chinese Society of Clinical Oncology (CSCO) guidelines for the diagnosis and treatment of NSCLC in 2019 indicated that the surgical indications for patients with stage IA, IB, IIA and IIB are relatively clear. However, the treatment plan for patients with stage III is still controversial. The treatment plan for patients with pleural and mediastinal invasion mainly includes chemotherapy, targeted therapy and immunotherapy. For inoperable NSCLC patients, we used sequencing of tissue samples to detect positive driver genes, through which treatment with targeted therapies was applied.^4^ Examples of these driver genes include EGFR mutation, ALK fusion, ROS1 fusion, BRAF V600E mutation/NTRK fusion and others.^5-7^ There has been an increasing evidence from clinical data that indicates the involvement of abnormal gene expressions and mutations in the occurrence and development of NSCLC. Gene mutations in NSCLC are more common in EGFR and ALK types. Among them, more studies are being conducted on EGFR tyrosine kinase inhibitors (EGFR-TKIs). With advancement in deep sequencing, the third generation of EGFR-TKIs has been developed and approved by the FDA in 2018. The polymorphism of EGFR-T790M has been found by many studies to increase the incidence of NSCLC in the Chinese population. The chronic stimulation of EGFR mutations plays a key role in tumor transformation and development of NSCLC. The ALK gene is a powerful oncogenic driver gene in NSCLC. It has been reported by many studies that the oncogenic EML4-ALK fusions and its increased DNA copy number are related to poor prognosis for patients with NSCLC. Despite the progress in NSCLC research, lack of effective diagnostic methods in the early stages of the disease and the increased tolerance of anti-tumor drugs in clinical treatment result in a very low five-year survival rate for NSCLC.^8^ In addition to the above-mentioned common gene mutations, there are many potential genes related to the prognosis and treatment of NSCLC, which need to be investigated. Therefore, it is very important to understand the precise molecular mechanisms related to the occurrence, development, proliferation, recurrence and prognosis of NSCLC. This will help to formulate effective diagnosis and treatment strategies, find potential targeted genes and provide more support for the clinical diagnosis and treatment.

 In the past few decades, in-depth research has been made on microarray technology and bioinformatic analysis. These technologies have been widely applied to genome-level screening research,^9^ which helps us to identify the differences in NSCLC. Nevertheless, the molecular mechanism and signal pathways of the occurrence and process of expressed genes remain poorly defined. Besides, false positive results often occur in independent microarray technology analysis, which makes it difficult to obtain reliable results.^10^ Therefore, in this study, three mRNA microarray gene datasets were retrieved from the Gene Expression Omnibus (GEO) and analyzed, and the differentially expressed genes (DEGs) between the NSCLC tissue and non-cancerous tissue were obtained. Subsequently, Gene Ontology (GO), Kyoto Encyclopedia of Genes and Genomes (KEGG) pathway enrichment analysis and protein-protein interaction (PPI) network analysis were performed to understand the molecular mechanisms of the occurrence and development of NSCLC. The identified DEGs may be candidate biomarkers for NSCLC, providing potential candidate gene selection for future targeted therapy of NSCLC.

## Materials and Methods

###  Microarray Data Processing

 GEO (http://www.ncbi.nlm.nih.gov/geo) is a public functional genomics database for high-throughput gene expression data, gene chips and microarrays. We retrieved 3 genome expression datasets from GEO (Aff or system GPL570 platform, Affymetrix Human Genome U133 Plus 2.0 Array) [(GSE10072),^11^ (GSE19188)^12^ and (GSE40791).^13^ We converted these genomic probes into the corresponding gene names according to the information provided in the platform. The GSE10072, GSE19188 and GSE40791 datasets contain 58, 91 and 94 NSCLC tissue samples and 49, 65 and 100 non-cancer tissue samples, respectively.

###  Screening and Identification of DEG

 GEO2R (http://www.ncbi.nlm.gov/geo2r)^14^ is an interactive network tool to compare and analyze two or more datasets in the GEO database in order to identify DEGs under experimental conditions. We used the GEO2R tool to screen DEGs between NSCLC and non-cancer samples. The adjusted *P* values and Benjamini and Hochberg false discovery rate were used to discover statistically significant hub genes and correct false positives, while probe sets without corresponding gene names or genes with multiple probe sets were removed. The values of adjusted *P* < 0.05, logFC > 1 were used to identify upregulated genes, and logFC < 1 was used to identify downregulated genes.^15,16^

###  PPI Network Construction and Module Analysis

 We used the STRING online database (http://string-db.org, version 10.0) to search for interacting genes to construct the PPI network.^15^ We used it to analyze the functional interactions between proteins, which can provide important information on the mechanism of disease occurrence and development. Cytoscape^16^ is an open-source bioinformatics software platform. It helps the users to visually analyze and construct networks of the genes of interest and integrate, analyze and visualize the data. As a result, it helps to achieve the purpose of analyzing the interactions between genetic and biological information. Cytoscape’s plug-in Molecular Complex Detection (MCODE)^17^ is based on topology-based network clustering to find densely connected areas. We used Cytoscape (version 3.7.2) to draw the PPI network and MCODE (version 1.6.1) to identify the most important modules in the PPI network. The selection criteria were as following: MCODE scores > 5, degree cut-off = 2, node score cut-off = 0.2, Max depth = 100 and k-score = 2.

###  Selection and Analysis of Hub Genes

 We investigated the functional roles of 10 hub genes with a degree ≥ 10. We used the cBioPortal (http://www.cbioportal.org)^18^ online platform to analyze gene networks and co-expressed genes. Then, we used the biological network gene oncology tool (BiNGO) (Version 3.0.4)^19^ in the Cytoscape plug-in to visualize the hub gene biological process. The UCSC Cancer Genomics Browser (http://genome-cancer.ucsc.edu)^20^ was used to construct a hierarchical cluster of the hub genes. Next, we used the Kaplan-Meier curve in cBioPortal to analyze the overall survival and disease-free survival of the hub genes and analyze other survival conditions that affect the prognosis of NSCLC, including tumor staging and grading, smoking status, etc. Finally, we used the online database Oncomine (http://www.oncomine.com)^21^ to analyze the expression pattern and tumor grade of the hub genes.

###  KEGG and GO Enrichment Analysis of Hub Gene

 The Database for Annotation, Visualization and Integrated Discovery (DAVID) (http://david.ncifcrf.gov, version 6.7)^22^ is an online biological information database that includes biological data and analysis tools. It provides a comprehensive extraction of the gene biological information and protein data and information. KEGG is an online database resource which is used to collect a large amount of molecular data generated through high-throughput experiments to understand the high-level and biological functions of the corresponding hub genes. GO is an analytical type of bioinformatics tool which is used to explain and analyze the biological processes of these genes. In order to better analyze the biological functions of the hub genes and DEGs, we used the DAVID online database and performed KEGG and GO analysis to achieve a deeper understanding of the biological characteristics of the occurrence and development of NSCLC.

###  Prognostic Analysis of Hub Gene

 The R software (version 4.0.2) is a collection of toolkits used for the annotation, processing, analysis and visualization of biological data. It consists of a series of packages.^23^ We retrieved our transcriptome data and the clinical group data of the screened hub genes in the TCGA database.^24^ Then, we ran the analysis using the R software to obtain the relevant mRNA expression of the mRNA of hub genes and map its corresponding risk curve, which represents the risk analysis of the prognostic survival time of NSCLC. The independent prognostic analysis is expressed in the form of forest diagrams, while the trend analysis is expressed by the receiver operating characteristic (ROC) curves. Single-factor and multi-factor Cox regression analysis indicate the clinical staging, and the risk score represents an independent prognostic factor.^25,26^

## Results

###  DEGs Screened and Identified

**Figure 1:**
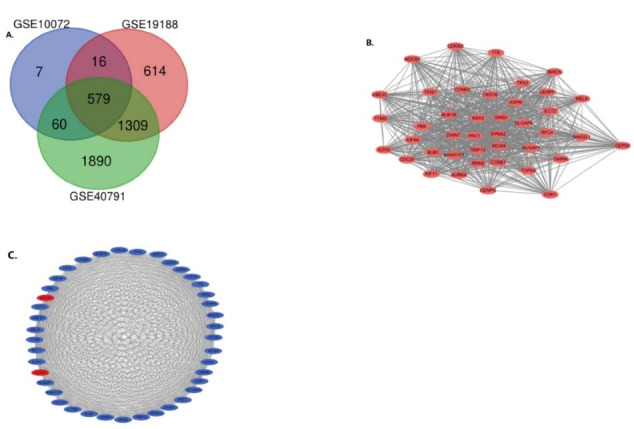
Venn Diagram and PPI Network of DEGs. A) DEGs were selected with a fold change > 2 and *P* value < 0.05 among the mRNA expression profiling sets GSE10072，GSE19188 and GSE40791. The three datasets showed an overlap of 579 genes. B) The PPI network of DEGs was constructed using Cytoscape. Upregulated genes are marked in light red, while downregulated genes are marked in light blue. C) The most significant module was obtained from PPI network with 41 nodes and 801 edges

 After obtaining the microarray normalization results, DEGs were screened and identified in three datasets: GSE10072 (859), GSE19188 (3657) and GSE40791 (6189). The overlap between the datasets contained 579 genes, as shown in the Venn diagram (Figure 1A), consisting of 343 upregulated genes and 236 downregulated genes in NSCLC tissues and non-cancerous tissues.

###  PPI Network Construction and Module Analysis

 We built the PPI network of DEGs using the Cytoscape software (Figure 1B) and used the Cytoscape plug-in MCODE to obtain the most important modules (Figure 1C). The results of function analysis of the hub genes^27^ using DAVID showed that the selected DEGs were mainly enriched in cell division, mitosis, nuclear division and cell cycle, as shown in Table 1.

**Table 1:** GO and KEGG Pathway Enrichment Analysis of DEGs in the Most Significant Module

**Term**	**Count**	**%**	***P*** ** value**	**FDR**
hsa04610:Complement and coagulation cascades	13	0.015564575	1. UE-05	0.014323205
hsa04512:ECM-receptor interaction	13	0.015564575	1.20E-04	0.154467703
hsa04974:Protein digestion and absorption	13	0.015564575	1.35E-04	0.172767737
hsaO4U5:p53 signaling pathway	9	0.010775475	0.00415009	5.202475569
hsa04510:Focal adhesion	17	0.020353675	0.006182138	7.657719001
hsa04110:Cell cycle	12	0.0143673	0.008516724	10.40605319
hsa05202:Transcriptional misregulation in cancer	14	0.01676185	0.012777216	15.22807973
hsa04151:PI3K-Akt signaling pathway	23	0.027537325	0.014475026	17.08206542
hsa05146:Amoebiasis	10	0.01197275	0.021524457	24.38706471
hsaO4514:Cell adhesion molecules	12	0.0143673	0.021828745	24.6885954
hsa04114:Oocyte meiosis	10	0.01197275	0.028056965	30.62192317
hsa04068:FoxO signaling pathway	11	0.013170025	0.035073485	36.78815863
hsa04270:Vascular smooth muscle contraction	10	0.01197275	0.037600738	38.88239916
hsaO5166:HTLV-I infection	17	0.020353675	0.03777001	39.02035566
hsa05144:Malaria	6	0.00718365	0.040274546	41.02828533
hsa03320:PPAR signaling pathway	7	0.008380925	0.044757247	44.47065359
hsa04914:Progesterone-mediated oocyte maturation	8	0.0095782	0.051422539	49.24770846
hsa05200:Pathways in cancer	23	0.027537325	0.05218087	49.76649163
hsa04614:Renin-angiotensin system	4	0.0047891	0.057734986	53.41962981
hsa04611:Platelet activation	10	0.01197275	0.065349769	58.03077189
hsa04670:Leukocyte transendothelial migration	9	0.010775475	0.077846058	64.69536318
hsa04925:Aldosterone synthesis and secretion	7	0.008380925	0.09381443	71.79207124

FDR, false discovery rate.

###  Hub Gene Selection and Analysis

 A total of 10 genes were identified as hub genes with a degree ≥ 10. The names, abbreviations and functions of these hub genes are shown in Table 2. A Kaplan-Meier curve^28^ was used to analyze the overall survival of the hub genes, among which CEP55, NMU, CAV1, TBX3, FBLN1 and SYNM had a *P* < 0.05, indicating that the expression of the seven hub genes selected from the NSCLC tissue samples has a certain significance on the prognosis and survival of the patients. In addition, the survival curve drawn using the three genes of SEMA6A, DMD and TPSB2 had a *P* > 0.05, indicating that the difference was not statistically significant. Subsequently, using the cBioPortal online platform to analyze the hub genes and perform co-expression analysis, the hub genes could basically distinguish between NSCLC and non-cancerous samples. The mRNA expression of the hub genes in the NSCLC samples as obtained from the TCGA database

**Table 2:** Functional Roles of 10 Hub Genes with Degree ≥ 10

**Gene Symbol**	**Full Name**	**Function**
CEP55	Centrosomal protein 55	Plays a role in mitotic exit and cytokinesis, recruits PDCD6IP and TSG101 to midbody during cytokinesis.
NMU	Neuromedin U	Stimulates muscle contractions of specific regions of the gastrointestinal tract. In humans, NMU stimulates contractions of the ileum and urinary bladder.
CAV1	Caveolin 1	Drives caveolae formation. Mediates the recruitment of CAVIN proteins (CAVIN1/2/3/4) to the caveolae.
SEMA6A	Semaphorin 6A	Cell surface receptor for PLXNA2 that plays an important role in cell-cell signaling. Transcriptional repressor involved in developmental processes.
TBX3	T-box transcription factor 3	Role in limb pattern formation. Acts as a negative regulator of PML function in cellular senescence. Incorporated into fibronectin-containing matrix fibers.
FBLN1	Fibulin 1	Adhesion and migration along protein fibers within the extracellular matrix (ECM).
SYNM	Synemin	Probable methyltransferase. Anchors the extracellular matrix to the cytoskeleton via F-actin. Tryptase is the major neutral protease present in mast cells and is secreted upon the coupled activation- degranulation response of this cell type. May play a role in innate immunity.
METTL7A	Methyltransferase like 7A	
DMD	Dystropliin	
TPSB2	Tryptase beta 2	

###  KEGG and GO Enrichment Analysis of Hub Genes

**Figure 2:**
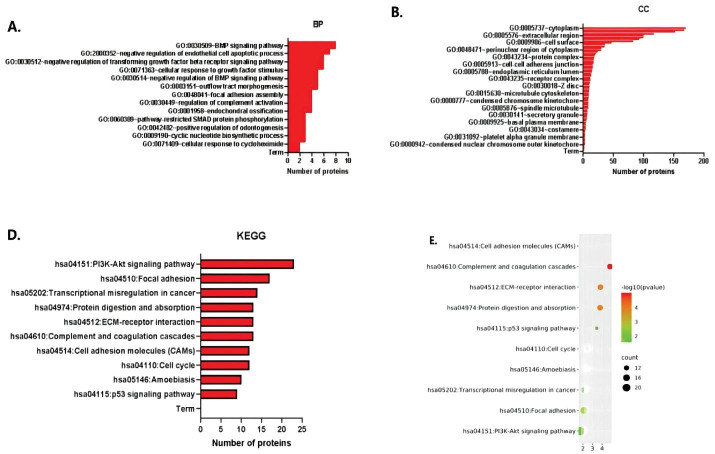
GO Enrichment Analysis and KEGG Enrichment Analysis of Key Genes. A) Histogram of BP Enrichment Analysis for Differential Genes in Lung cancer. B) Histogram of CC Enrichment Analysis for Differential Genes in Lung cancer. C) Histogram and bubble diagram of KEGG enrichment analysis of different genes in lung cancer. D) KEGG pathway enrichment analysis bubble diagram

 The function and pathway enrichment analysis of hub genes was performed using DAVID. The results of GO analysis showed that the changes in the biological process (BP) terms of the hub genes^29^ were significantly increased in signal pathways, regulation of cell division, regulation of complement activation and mitotic cell cycle (Figure 2A). On the other hand, the changes in the cell component (CC) terms^30^ were mainly concentrated in the cytoplasmic area, extracellular area of the cell membrane, area around the nucleus of the cytoplasm, concentrated chromosomal centromeres and platelets in the hyaluronic acid granules (Figure 2B). The GO analysis showed that function of the hub genes mainly focuses on carbohydrate binding, signal transduction activity, protein dimerization activity regulation, phosphotransferase activity regulation, DNA specific binding regulation, transcription activator activity, RNA polymerase II core promoter and others. The KEGG pathway analysis showed that the hub genes are involved in the PI3K-Akt signaling pathway, protein cascade activation, ECM receptor interaction, complement and coagulation cascade activation, cell adhesion molecules, cell division cycle and p53 tumor suppressor gene regulation (Figures 2C and 2D).

###  Survival Prognosis Analysis of Hub Gene

####  Risk Analysis

**Figure 3:**
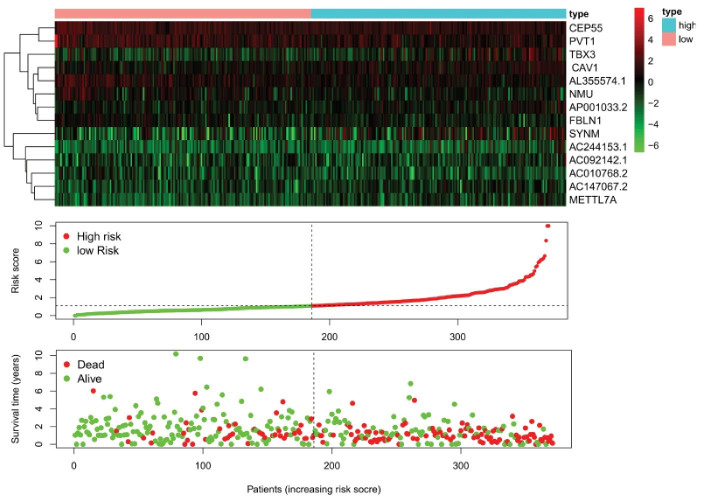
Survival Curve Analysis of the Expression of Hub Genes and Tumor Grade in the TCGA Lung Dataset. Hub genes mRNA expression in LCA compared to normal lung tissues

**Figure 4:**
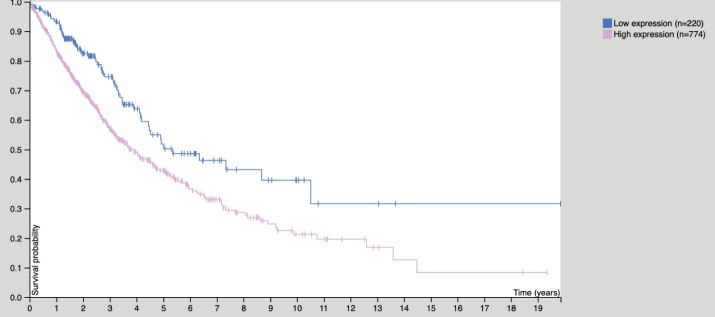
The expression of CEP55 affects the survival curve of NSCLC

 We downloaded the clinical prognostic data of NSCLC from the TCGA database, divided these patients into high-risk and low-risk groups and used R language to draw a risk curve.^31^ The risk curve and the survival status diagram were drawn using the same sample data (Figure 3). On the survival status chart, we can observe that from left to right, the patient’s risk increases in sequence, the patient’s survival time also appears to decrease in sequence, and the mortality increases, which is in line with the expected results (Figure 4).

####  Independent Prognostic Analysis

**Figure 5:**
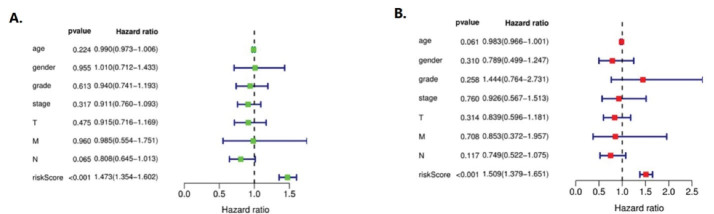
Independent Survival and Prognosis Analysis. (A) A single-factor independent prognostic analysis of NSCLC. (B) A multifactorial independent prognostic analysis of NSCLC

 As shown in Figure 5, A is the univariate independent prognostic analysis of CEP55 expression and clinical features of NSCLC, and B is the multivariate independent prognostic analysis of CEP55 expression and clinical features of NSCLC.

 In univariate analysis, CEP55 expression in patients with NSCLC was unrelated to age (HR = 0.990, 0.973 to 1.006, *P* = 0.224), gender (HR = 1.010, 0.712–1.433, *P* = 0.9555), grade (HR = 0.940, 0.741–1.193, *P* = 0.613), stage (HR = 0.911, 0.760–1.093, *P* = 0.317), T (HR = 0.915, 0.716 ~ 1.169, *P* = 0.475), N (HR = 0.808, 0.645 ~ 1.013, *P* = 0.065), M (HR = 0.985, 0.554 ~ 1.751, *P* = 0.960). It was correlated with risk score (HR = 1.473, 1.354–1.602, *P* < 0.001), indicating that CEP55 was a high risk gene of risk score.

 In multivariate analysis, CEP55 expression was associated with age of patients with NSCLC (HR = 0.983, 0.966~1.001, *P* = 0.061), gender (HR = 0.789, 0.499~1.247, *P* = 0.310), grade (HR = 1.444, 0.764~2.731, *P* = 0.258), stage (HR = 0.926, 0.567~1.513, *P* = 0.760), T (HR = 0.839, 0.596~1.181, *P* = 0.314), N (HR = 0.749, 0.522~1.075, *P* = 0.117), M (HR = 0.853, 0.372–1.957, *P* = 0.708). It was correlated with risk score (HR = 1.509, 1.379–1.651, *P* < 0.001), which also indicated that CEP55 was a high risk gene in risk score, and its statistical results were consistent with the results of univariate analysis.

####  Multi-index ROC Curve

**Figure 6:**
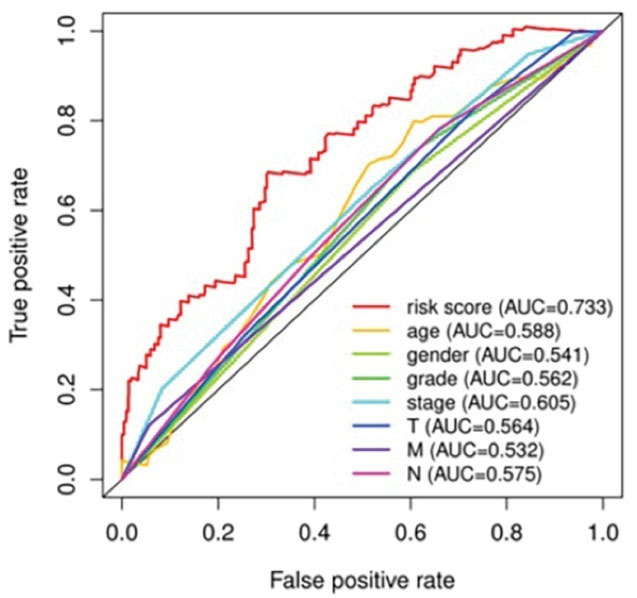
Multi-indicator ROC curve for NSCLC (The abscissa of ROC curve is false positive rate, and the ordinate is true positive rate)

 The purpose of using the R software to construct a multi-index ROC curve is to judge and evaluate the accuracy of the NSCLC prognostic model.^33^ The ROC curve is the risk value of the NSCLC model we constructed. The area of the curve is between 0.5 and 1.0. If the risk score area under the curve (AUC) > 0.90, it means that this prognostic model is very accurate in predicting the survival time of the patient. If the AUC is from 0.70 to 0.90, it means that this prognostic model has relatively high accuracy in predicting the survival time of the patient. If the AUC is from 0.50 to 0.70, it means that this prognostic model can be used to predict the patient’s survival time. As shown in Figure 6, our model risk score (AUC = 0.733) shows that the NSCLC model we built can accurately predict the survival time of patients, and the risk value prediction of our application of this model is better than other clinical traits.

###  Experimental Verification

**Figure 7:**
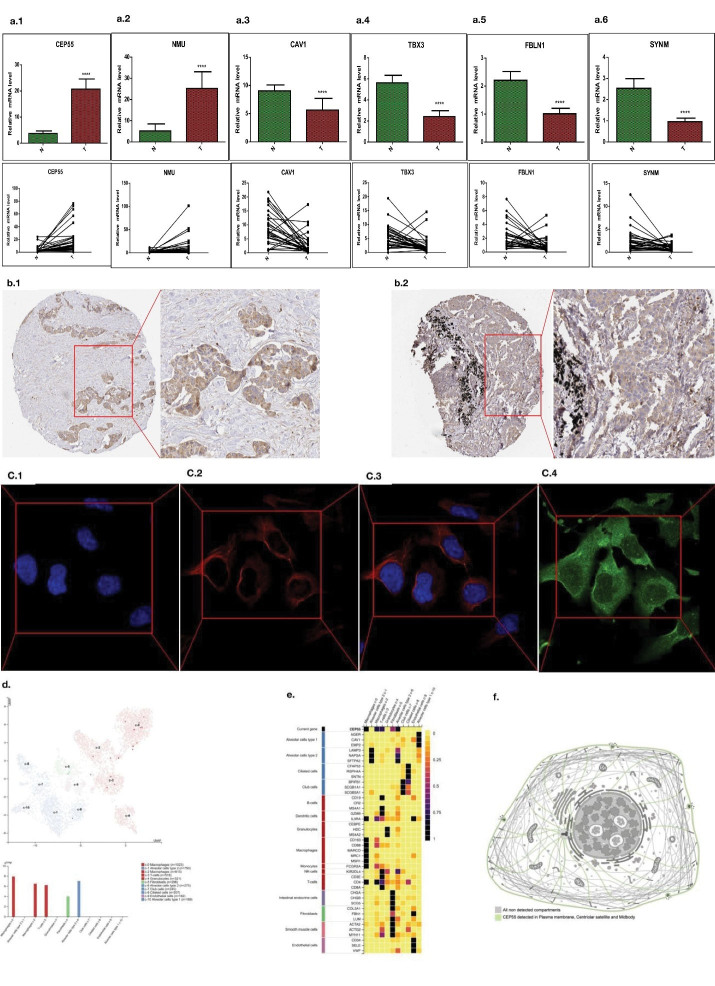
Experimental Verification of Lung Adenocarcinoma Tissue. (a1-a6): The expression of prognostic genes in 32 lung cancer patients was verified by qRT-PCR assay (N, normal tissue; T, tumor tissue). (b1 & b2) The relationship between CEP55 expression level and clinicopathological features of lung adenocarcinoma patients. (c1-c4) Cellular immunofluorescence of CEP55. (d) RNA sequencing map of CEP55 in lung cancer tissue. (e) Correlation of CEP55 expression and aggregation heat map of various immune cells. (f) mRNA expression level of CEP55 in lung adenocarcinoma tissues

 In this study, the high and low expression of related genes in the lung adenocarcinoma tissues and paracancerous tissues was verified through the polymerase chain reaction (PCR) experiments. Real-time fluorescence quantitative PCR generally presents the results in the form of mean ± standard deviation. We utilized 32 lung cancer specimens, including lung cancer tissues and adjacent tissues, and verified using quantitative real-time PCR (qRT-PCR). The experimental results (Figure 7) show that CEP55, NMU, CAV1, TBX3, FBLN1and SYNM have significantly different expression in lung cancer. These genes can have a prognostic value of NSCLC, including two upregulated genes (CEP55 and NMU) and five downregulated ones (CAV1, TBX3, FBLN1 and SYNM) (Supplementary file).

 The newly discovered centrosomal protein CEP55 (55 kD) is a member of the coiled-coil protein family. Its main function is to anchor microtubule aggregation related proteins, which participate in spindle formation and regulate cell proliferation. This protein is expressed in both normal tissues and tumor cells, and binds to centrosomes and intermediates in the cell cycle. After phosphorylation, it plays a role in regulating the cell cycle.^30^ It has been found that CEP55 overexpression is significantly correlated with tumor stage, invasiveness and metastasis of many malignant tumors. Subsequent studies described how CEP55 works with members of the ESCRT family to contract the intracellular bridge and ultimately lead to cell division.^31^ CEP55 plays an important role in intermediate-dependent cell functions such as centrosome replication, cell cycle progression, and cytokinesis.

 Recently, high expression of CEP55 has been found in many human tumors, and high expression of CEP55 is associated with malignancy, invasion and poor prognosis. Xie et al^32^ found that CEP55 expression was up-regulated in lung cancer by PCR and immunohistochemistry. To Duc^33^ analyzed the gene expression microarray map of lung cancer tissues and adjacent tissues as well as between metastatic and primary foci, and found that CEP55 expression was abnormally increased in lung adenocarcinoma, and was closely related to the metastasis and invasion ability of cancer cells.

## Discussion

 NSCLC is one of the most common malignant tumors in the world and the main cause of malignancy-related death in China.^34^ The incidence of NSCLC has been increasing in recent years. Approximately 75% of the patients are at an advanced stage of the tumor when they are initially diagnosed, and the 5-year survival rate is very low. This has attracted many researchers to work on the understanding of the pathogenesis, diagnosis, treatment and follow-up of this cancer. However, the molecular mechanism of NSCLC is still poorly understood, and cell cycle regulators play a very important role in it.^35^ According to previous studies, the overexpression of CEP55, NMU, CAV1, TBX3, FBLN1, SYNM and METTL7A genes is related to the survival and prognosis of NSCLC patients.^36^

 In this study, three mRNA microarray datasets were analyzed to obtain the DEGs and hub genes between NSCLC tissues and non-tumor tissues. A total of 579 differential genes and 6 valuable hub gene were identified. Downregulated genes were mainly enriched in the processes of protein activation cascade, complement activation and so on. Zou et al^37^ found that the mitotic regulation disorder during the cell cycle plays an important role in the process of colorectal cancer formation. Liu et al^38^ investigated the tumor growth and anti-tumor therapy and reported that the activation of complement can promote the growth and proliferation of tumor cells. In addition, Zhang et al^39^ reported that the P53 gene plays a major role in the body’s anti-tumor process, participating in tumor cell antigen recognition, signal transmission and regulating apoptosis. The conclusions reported in these related reports are consistent with our research results. CEP55 is a member of the coiled-coil protein family. Its main function is to participate in the formation of the spindle by anchoring microtubule polymerization-related proteins, thereby regulating cell proliferation. It has been reported that the overexpression of CEP55 is significantly correlated with tumor staging, aggressiveness and metastasis of many malignant tumors.^40^ The overexpression of CEP55 has been found in NSCLC, pancreatic cancer, breast cancer and prostate cancer. CEP55 can participate in the regulation of cell cycle together with apoptin. The phosphorylation of PLK1 of CEP55 inhibits interaction with MKLP1, which leads to cell development and death. NMU is mainly the MC3/4R signal pathway to regulate the body’s energy balance. There are relatively few studies on NMU related research in the field of tumors. Some studies have reported that the NMU protein is also involved in the metabolic process of tumor cells.^41^ Similar to NMU, CAV1 participates in the energy metabolism and growth and development of tumor cells through metabolic pathways.^42^ TBX3 is a transcription inhibitor that regulates growth and development and is involved in the growth and division of tumor cells.^43^ FBLN1 is related to cell senescence. The mutation of FBLN1 gene prevents the tumor cells from senescence, which conforms to their growth characteristics. SYNM is an important gene protein that is downregulated during the carcinogenesis of colorectal cancer. It is prone to missense mutations in colorectal cancer. By analyzing the STRING database, we obtained the interaction proteins of SYNM and learned its main biological functions, represented in the process of combining cytoskeleton protein and actin, connecting between cells and between the cell and substrates. In addition, the DNA methylation of the SYNM gene can regulate the transcription level in thyroid cancer.

 Finally, we used a multi-index ROC curve to verify the NSCLC prognosis model constructed by the hub gene. The AUC = 0.733 suggests high prediction and evaluation accuracy of the survival time of NSCLC patients using this model.

 The contributions of this study are as follows: (1) Instead of the traditional single GEO to perform data analysis of GEGs, we combined multiple CEO databases to reduce the deviation of the research results. (2) We explored the KEGG and GO enrichment analysis and signaling pathways of DEGs gene and hub genes in the lung cancer prognostic model. (3) We built a more convincing prognostic model of lung cancer by combining the clinical trial data and gene transcription data. (4) The prognosis and 5-year survival rate of the patients with NSCLC were evaluated by independent prognostic analysis of its single factor and multiple factors, and the risk value of NSCLC patients was calculated. The screening and identification of DEGs genes and hub genes will provide guidance for the clinical treatment of patients with lung cancer. In the future, further genes that have not been deeply explored may become potential treatment sites for targeted therapy of lung cancer.

 This study suffered from some limitations: (1) The data downloaded from the CEO database did not strictly subdivide the pathological types of lung cancer; thus, it did not distinguish between squamous cell carcinoma and adenocarcinoma of the lung. (2) The gene transcription data are sufficient, but more clinical data are needed for clinical sample data verification. (3) The analysis of hub genes could not deeply analyze the relevant signal pathways and mechanisms, which need to be verified by more basic experiments.^44^

 In conclusion, this study was conducted to identify and screen the DEGs and hub genes that may be involved in the carcinogenesis of NSCLC.Continued discovery of new roles of CEP55 and the different modulations of CEP55 in different types of cancer suggest that despite many advances in the study of CEP55, a thorough understanding of the physiological and pathological roles of CEP55 remains to be achieved.^45^ Changes in the expression and function of CEP55 during the development of human tumors may have some effects on the prognosis of patients with malignant tumors with overexpression of CEP55. With the deepening of the research, it will bring more scientific basis for the treatment of cancer, which will have a profound and meaningful impact.

## Supplementary files

Supplementary file 1 contains Table S1.
