# Supplementary material for: The Role of CEP55 Expression in Tumor Immune Response and Prognosis of Patients with Non-small Cell lung Cancer
Source: Arch Iran Med. 2022 Jul 1;25(7):432–42. doi: 10.34172/aim.2022.72 (PMC11904281; doi:10.34172/aim.2022.72)

sample		ΔCt	ΔΔCt	fold change	ΔCt	ΔΔCt	fold change	ΔCt	ΔΔCt	fold change	ΔCt	ΔΔCt	fold change	ΔCt	ΔΔCt	fold change	ΔCt	ΔΔCt	fold change	ΔCt	ΔΔCt	fold change
		CEP55			NMU			CAV1			TBX3			FBLN1			SYNM			METTL7A		
test		7.515	-4.33	20.11221	14.045	0	1	3.63	0	1	8.06	0	1	4.61	0	1	6.95	0	1	5.17	0	1
0124RT-N2		11.845	0	1	12.375	-1.67	3.182146	3.45	-0.18	1.132884	7.195	-0.865	1.82134	2.035	-2.575	5.95871	6.275	-0.675	1.596597	4.095	-1.075	2.106722
0124RT-N3		9.45	-2.395	5.259771	13.64	-0.405	1.324089	-0.615	-4.245	18.96148	4.29	-3.77	13.64216	3.15	-1.46	2.751084	5.555	-1.395	2.629886	3.685	-1.485	2.799172
0129RT3-N5		11.325	-0.52	1.433955	14.655	0.61	0.655197	1.86	-1.77	3.41054	6.545	-1.515	2.857988	5.645	1.035	0.488016	5.895	-1.055	2.077718	5.6	0.43	0.742262
0129RT3-N6		11.425	-0.42	1.337928	15.195	1.15	0.450625	0.995	-2.635	6.211751	6.045	-2.015	4.041806	3.94	-0.67	1.591073	5.835	-1.115	2.16595	4.275	-0.895	1.85961
0129RT3-N7		11.835	-0.01	1.006956	16.385	2.34	0.19751	0.87	-2.76	6.773962	5.505	-2.555	5.876675	4.235	-0.375	1.29684	6.685	-0.265	1.201636	4.535	-0.635	1.552938
0129RT2-N9		11.26	-0.585	1.500039	15.475	1.43	0.371131	1.225	-2.405	5.296356	5.395	-2.665	6.342273	4.615	0.005	0.99654	5.795	-1.155	2.226843	4.13	-1.04	2.056228
0129RT1-N2		8.625	-3.22	9.317869	10.535	-3.51	11.3924	0.53	-3.1	8.574188	7.6	-0.46	1.375542	5.605	0.995	0.501736	6.72	-0.23	1.172835	6.48	1.31	0.403321
0129RT1-N3		11.175	-0.67	1.591073	16.37	2.325	0.199575	0.36	-3.27	9.646463	5.09	-2.97	7.835362	4.67	0.06	0.959264	4.395	-2.555	5.876675	4.775	-0.395	1.314943
0129RT1-N4		11.755	-0.09	1.06437	13.41	-0.635	1.552938	0.66	-2.97	7.835362	4.865	-3.195	9.157793	4.01	-0.6	1.515717	5.67	-1.28	2.42839	4.445	-0.725	1.652901
0129RT1-N6		11.39	-0.455	1.370783	17.755	3.71	0.076415	0.055	-3.575	11.91742	4.985	-3.075	8.426888	3.98	-0.63	1.547565	7.025	0.075	0.949342	4.145	-1.025	2.034959
0129RT1-N8		9.54	-2.305	4.941675	12.695	-1.35	2.549121	1.165	-2.465	5.521269	6.105	-1.955	3.877159	4.28	-0.33	1.257013	6.79	-0.16	1.117287	4.305	-0.865	1.82134
0129RT2-N1		11.2	-0.645	1.563739	14.69	0.645	0.639493	-0.47	-4.1	17.14838	6.07	-1.99	3.97237	4.41	-0.2	1.148698	6.7	-0.25	1.189207	4.035	-1.135	2.196186
0129RT2-N2		11.225	-0.62	1.536875	15.15	1.105	0.464902	0.865	-2.765	6.79748	6.78	-1.28	2.42839	4.415	-0.195	1.144724	6.46	-0.49	1.404445	5.23	0.06	0.959264
0129RT2-N3		11.91	0.065	0.955945	13.02	-1.025	2.034959	0.295	-3.335	10.09102	5.69	-2.37	5.169411	3.36	-1.25	2.378414	6.89	-0.06	1.042466	4.19	-0.98	1.972465
0129RT2-N4		11.93	0.085	0.942785	15.805	1.76	0.295248	0.705	-2.925	7.594737	5.675	-2.385	5.223439	4.78	0.17	0.888843	6.705	-0.245	1.185093	4.785	-0.385	1.30586
0129RT2-N5		10.815	-1.03	2.042024	12.205	-1.84	3.5801	0.215	-3.415	10.66639	5.29	-2.77	6.821079	2.285	-2.325	5.010658	5.08	-1.87	3.655326	3.865	-1.305	2.470837
0129RT2-N6	N	9.185	-2.66	6.32033	13.89	-0.155	1.113422	-0.125	-3.755	13.50105	4.965	-3.095	8.544523	2.865	-1.745	3.351949	5.82	-1.13	2.188587	3.2	-1.97	3.917681
0129RT2-N7		14.985	3.14	0.11344	14.775	0.73	0.602904	3.27	-0.36	1.283426	14.435	6.375	0.012049	4.66	0.05	0.965936	5.01	-1.94	3.837056	6.375	1.205	0.433769
0129RT2-N8		10.62	-1.225	2.337554	13.34	-0.705	1.630145	0.995	-2.635	6.211751	6.405	-1.655	3.149232	3.265	-1.345	2.540302	7.895	0.945	0.51943	4.735	-0.435	1.351911
newRT4-N1		10.655	-1.19	2.281527	15.635	1.59	0.332171	0.01	-3.62	12.295	6.705	-1.355	2.557971	3.59	-1.02	2.027919	6.94	-0.01	1.006956	5.085	-0.085	1.060688
newRT4-N2		10.395	-1.45	2.732081	10.985	-3.06	8.339726	0.11	-3.52	11.47164	4.8	-3.26	9.57983	3.145	-1.465	2.760635	5.59	-1.36	2.566852	3.435	-1.735	3.328795
newRT4-N3		11.255	-0.59	1.505247	13.72	-0.325	1.252664	0.755	-2.875	7.336032	4.88	-3.18	9.063071	3.26	-1.35	2.549121	4.03	-2.92	7.568461	4.32	-0.85	1.802501
newRT4-N4		12.26	0.415	0.750019	14.43	0.385	0.765779	3.96	0.33	0.795536	7.35	-0.71	1.635804	3.175	-1.435	2.703822	7.195	0.245	0.843816	4.745	-0.425	1.342573
newRT4-N5		10.225	-1.62	3.07375	13.82	-0.225	1.168777	-0.285	-3.915	15.08455	5.5	-2.56	5.897077	4.855	0.245	0.843816	6.37	-0.58	1.494849	4.955	-0.215	1.160704
newRT4-N6		10.775	-1.07	2.099433	#DIV/0!	#DIV/0!	#DIV/0!	-0.565	-4.195	18.31559	6	-2.06	4.169863	5	0.39	0.76313	5.185	-1.765	3.39874	5.235	0.065	0.955945
newRT4-N7		10.54	-1.305	2.470837	11.895	-2.15	4.438278	-0.815	-4.445	21.78103	3.78	-4.28	19.42712	2.205	-2.405	5.296356	3.3	-3.65	12.55335	3.245	-1.925	3.797368
newRT3-N1		7.25	-4.595	24.16756	7.615	-6.43	86.22295	-0.65	-4.28	19.42712	5.685	-2.375	5.187358	1.67	-2.94	7.674113	8.56	1.61	0.327598	2.69	-2.48	5.578975
		11.18	-0.665	1.585568	13.165	-0.88	1.840375	1.44	-2.19	4.563055	6.665	-1.395	2.629886	3.995	-0.615	1.531558	4.94	-2.01	4.027822	4.955	-0.215	1.160704
		8.925	-2.92	7.568461	13.01	-1.035	2.049114	1.91	-1.72	3.294364	6.76	-1.3	2.462289	4.36	-0.25	1.189207	7.3	0.35	0.784584	4.955	-0.215	1.160704
		10.465	-1.38	2.602684	#DIV/0!	#DIV/0!	#DIV/0!	0.775	-2.855	7.235035	5.16	-2.9	7.464264	2.53	-2.08	4.228072	4.875	-2.075	4.213444	4.11	-1.06	2.084932
		11.985	0.14	0.907519	#DIV/0!	#DIV/0!	#DIV/0!	1.66	-1.97	3.917681	5.7	-2.36	5.133704	4.455	-0.155	1.113422	5.33	-1.62	3.07375	5.555	0.385	0.765779
test		10.655	-1.19	2.281527	12.47	-1.575	2.979355	0.305	-3.325	10.02132	4.5	-3.56	11.79415	2.195	-2.415	5.333195	5.03	-1.92	3.784231	3.765	-1.405	2.648178
0124RT-T2		5.585	-6.26	76.63864	11.75	-2.295	4.90754	4.305	0.675	0.626332	7.635	-0.425	1.342573	4.99	0.38	0.768438	8.895	1.945	0.259715	4.81	-0.36	1.283426
0124RT-T3		7.85	-3.995	15.94464	9.355	-4.69	25.81254	1.25	-2.38	5.205367	5.6	-2.46	5.502167	4.895	0.285	0.820742	6.46	-0.49	1.404445	4.58	-0.59	1.505247
0129RT3-T5		7.635	-4.21	18.50701	9.615	-4.43	21.55574	5.57	1.94	0.260616	7.85	-0.21	1.156688	5.365	0.755	0.592546	8.265	1.315	0.401925	5.79	0.62	0.650671
0129RT3-T6		8.85	-2.995	7.972322	10.68	-3.365	10.30305	5.165	1.535	0.345079	7.065	-0.995	1.993081	5.635	1.025	0.49141	8.115	1.165	0.445964	5.71	0.54	0.687771
0129RT3-T7		6.31	-5.535	46.36615	4.46	-9.585	768.02	-0.485	-4.115	17.3276	11.06	3	0.125	3.525	-1.085	2.121375	#DIV/0!	#DIV/0!	#DIV/0!	0.79	-4.38	20.82147
0129RT2-T9		9.68	-2.165	4.484664	8.325	-5.72	52.70983	3.695	0.065	0.955945	7.065	-0.995	1.993081	5.895	1.285	0.410371	6.88	-0.07	1.049717	4.89	-0.28	1.214195
0129RT1-T2		7.275	-4.57	23.75238	10.31	-3.735	13.31518	1.4	-2.23	4.69134	7.085	-0.975	1.965641	3.77	-0.84	1.79005	6.09	-0.86	1.815038	5.07	-0.1	1.071773
0129RT1-T3		12.005	0.16	0.895025	14.155	0.11	0.926588	3.205	-0.425	1.342573	7.995	-0.065	1.046085	5.52	0.91	0.532185	6.905	-0.045	1.031683	5.935	0.765	0.588453
0129RT1-T4		6.32	-5.525	46.04587	7.38	-6.665	101.4764	6.175	2.545	0.171348	10.43	2.37	0.193446	6.185	1.575	0.335643	7.62	0.67	0.628507	8.515	3.345	0.098413
0129RT1-T6		9.975	-1.87	3.655326	11.405	-2.64	6.233317	3.955	0.325	0.798298	9.28	1.22	0.429283	5.045	0.435	0.739694	8.54	1.59	0.332171	4.31	-0.86	1.815038
0129RT1-T8		7.325	-4.52	22.94328	5.815	-8.23	300.2457	3.11	-0.52	1.433955	7.69	-0.37	1.292353	5.86	1.25	0.420448	7.925	0.975	0.50874	5.635	0.465	0.724471
0129RT2-T1		9.92	-1.925	3.797368	12.835	-1.21	2.313376	2.09	-1.54	2.907945	4.195	-3.865	14.57072	2.645	-1.965	3.904127	5.13	-1.82	3.530812	4.54	-0.63	1.547565
0129RT2-T2	T	9.115	-2.73	6.634556	11.66	-2.385	5.223439	3.315	-0.315	1.244012	8.19	0.13	0.913831	5.225	0.615	0.65293	7.675	0.725	0.604997	7.03	1.86	0.275476
0129RT2-T3		10.315	-1.53	2.887858	11.74	-2.305	4.941675	1.99	-1.64	3.116658	7.275	-0.785	1.723092	4.585	-0.025	1.01748	8.35	1.4	0.378929	5.205	0.035	0.976032
0129RT2-T4		9.44	-2.405	5.296356	11.66	-2.385	5.223439	4.215	0.585	0.666649	8.385	0.325	0.798298	5.83	1.22	0.429283	8.98	2.03	0.244855	6.26	1.09	0.469761
0129RT2-T5		8.335	-3.51	11.3924	14.77	0.725	0.604997	4.14	0.51	0.702222	9.61	1.55	0.34151	6.545	1.935	0.261521	7.895	0.945	0.51943	6.67	1.5	0.353553
0129RT2-T6		6.005	-5.84	57.2816	10.56	-3.485	11.19669	4.72	1.09	0.469761	7.15	-0.91	1.879045	7.62	3.01	0.124137	8.595	1.645	0.319746	7.475	2.305	0.202361
0129RT2-T7		7.35	-4.495	22.54913	6.925	-7.12	139.1021	2.925	-0.705	1.630145	8.355	0.295	0.815072	6.48	1.87	0.273573	6.3	-0.65	1.569168	7.195	2.025	0.245705
0129RT2-T8		7.92	-3.925	15.18947	11.335	-2.71	6.543216	2.07	-1.56	2.948538	7.225	-0.835	1.783857	4.915	0.305	0.809442	7.295	0.345	0.787308	5.22	0.05	0.965936
newRT4-T1		8.38	-3.465	11.04254	12.87	-1.175	2.257929	0.16	-3.47	11.08088	8.035	-0.025	1.01748	5.275	0.665	0.630689	8.345	1.395	0.380245	5.87	0.7	0.615572
newRT4-T2		8.535	-3.31	9.917662	12.795	-1.25	2.378414	-1.575	-5.205	36.88596	6.625	-1.435	2.703822	4.81	0.2	0.870551	6.69	-0.26	1.197479	4.915	-0.255	1.193336
newRT4-T3		8.71	-3.135	8.784743	9.595	-4.45	21.85664	-2.115	-5.745	53.63118	8.035	-0.025	1.01748	5.735	1.125	0.458502	7.775	0.825	0.564482	6.38	1.21	0.432269
newRT4-T4		5.665	-6.18	72.50457	12.545	-1.5	2.828427	0.745	-2.885	7.387058	7.265	-0.795	1.735077	5.005	0.395	0.760489	8.805	1.855	0.276433	4.875	-0.295	1.226885
newRT4-T5		8.655	-3.19	9.12611	10.22	-3.825	14.17228	1.555	-2.075	4.213444	5.95	-2.11	4.316913	5.5	0.89	0.539614	6.34	-0.61	1.526259	5.215	0.045	0.96929
newRT4-T6		9.24	-2.605	6.083915	11.62	-2.425	5.37029	4.75	1.12	0.460094	7.47	-0.59	1.505247	4.905	0.295	0.815072	7.08	0.13	0.913831	4.645	-0.525	1.438934
newRT4-T7		8.505	-3.34	10.12605	11.165	-2.88	7.361501	3.705	0.075	0.949342	6.275	-1.785	3.446185	4.41	-0.2	1.148698	7.34	0.39	0.76313	4.145	-1.025	2.034959
newRT3-T1		7.21	-4.635	24.847	6.9	-7.145	141.5335	4.13	0.5	0.707107	7.635	-0.425	1.342573	6.555	1.945	0.259715	8.02	1.07	0.476319	6.005	0.835	0.560583
		#DIV/0!	#DIV/0!	#DIV/0!	#DIV/0!	#DIV/0!	#DIV/0!	#DIV/0!	#DIV/0!	#DIV/0!	#DIV/0!	#DIV/0!	#DIV/0!	#DIV/0!	#DIV/0!	#DIV/0!	#DIV/0!	#DIV/0!	#DIV/0!	#DIV/0!	#DIV/0!	#DIV/0!
		5.77	-6.075	67.41511	8.465	-5.58	47.83518	4.175	0.545	0.685391	8.125	0.065	0.955945	5.23	0.62	0.650671	9.155	2.205	0.216885	9.195	4.025	0.061426
		7.855	-3.99	15.88948	10.54	-3.505	11.35299	5.03	1.4	0.378929	7.39	-0.67	1.591073	4.235	-0.375	1.29684	7.925	0.975	0.50874	5.195	0.025	0.982821
		8.28	-3.565	11.8351	8.065	-5.98	63.11889	2.83	-0.8	1.741101	6.21	-1.85	3.605002	3.605	-1.005	2.006943	5.725	-1.225	2.337554	3.885	-1.285	2.436821

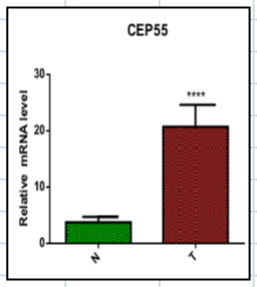

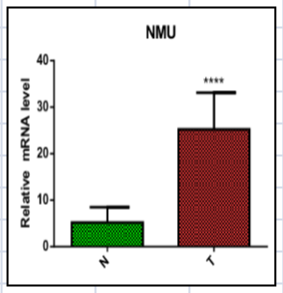

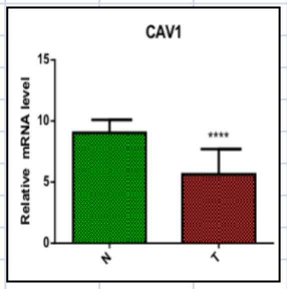

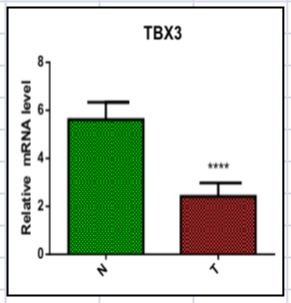

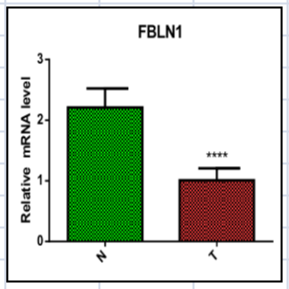

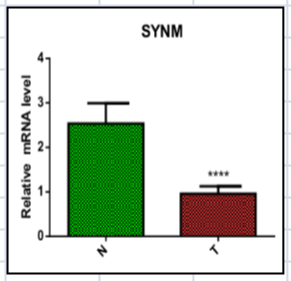

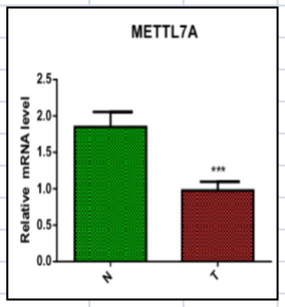

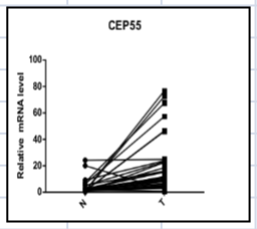

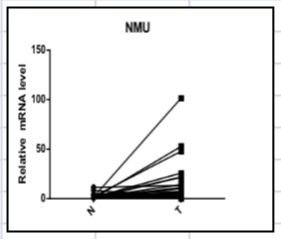

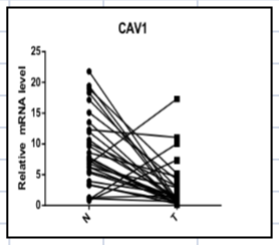

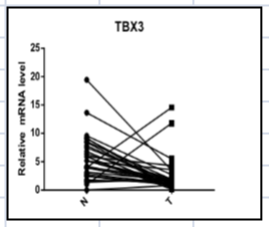

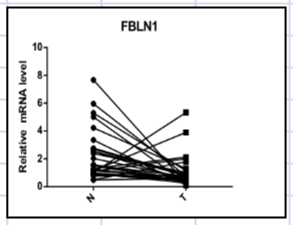

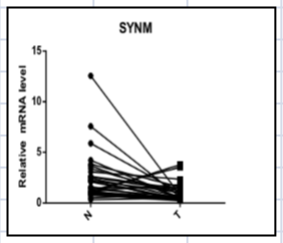

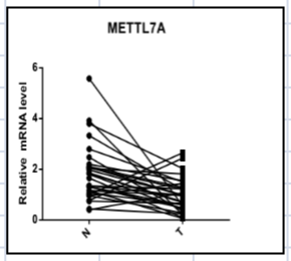

Supplement: Supplementary file 1 — contains Table S1. [file aim-25-432-s001.pdf]
